# A new species of *Corydalus* Latreille from Venezuela (Megaloptera, Corydalidae)

**DOI:** 10.3897/zookeys.67.702

**Published:** 2010-11-10

**Authors:** Atilano Contreras-Ramos, Klaus von der Dunk

**Affiliations:** 1 Instituto de Biología, UNAM, Depto. de Zoología, Apdo. Postal 70-153, 04510 México, D.F., México; 2 Kreis Nürnberger Entomologen, Ringstraße 62, 91334 Hemhofen, Germany

**Keywords:** Dobsonfly, taxonomy, biodiversity, South America, key

## Abstract

A new species of dobsonfly, Corydalus wanningeri, from Venezuela, is described and illustrated. It superficially resembles Corydalus neblinensis Contreras-Ramos, with a uniform reddish coloration of body and wings. Yet, because of male genitalic structure it might be closely related to Corydalus crossi Contreras-Ramos. Specimens were collected from a rain forest transitional zone between the Orinoco lowlands and the Gran Sabana plateau, in Bolívar state. This is the 15^th^ species of Corydalus to be recorded from Venezuela, rendering this the country with the highest number of documented Corydalus species. A key to the sexually dimorphic, long-mandibled Venezuelan species of the genus is provided.

## Introduction

The dobsonfly genus Corydalus Latreille was revised nearly a decade ago (Contreras-Ramos 1998), with 30 species recognized. Four species from Venezuela were later added to the genus (Contreras-Ramos 2002), and a 35th species is herein described, also from Venezuela. Corydalus is the most species rich genus of New World dobsonflies, a monophyletic lineage also including Chloronia Banks and Platyneuromus Weele. Most Corydalus species, 27 (77%), are South American only, and three are South and Central American. Fifteen species (43%) have been recorded from Venezuela, of which seven (20%), or possibly eight, are known only from this country (Table 1). Brazil rates second, with 11 species recorded (31%), of which only three (9%), or possibly four, are uniquely recorded for the country. These numbers indicate a pattern of high species richness and strong endemicity for dobsonflies in Venezuela.

Late last year the authors established contact. From images of a collection of Neotropical dobsonflies, a series of Corydalus specimens stood out as potentially new, a supposition corroborated upon specimen examination. Specimens of the new species had been collected by Professor Rupert Wanninger of Bavaria, where he is now a retired teacher of arts and sports. Prof. Wanninger is also a recognized amateur entomologist, deeply versed in Natural History, with extensive experience in breeding of exotic beetles. For years, he has motivated young people in an interest and respect for Nature. This contribution is in homage to Prof. Wanninger’s lifetime as an educator and insect lover.

**Table:** **Table 1.** Species of Corydalus Latreille recorded from Venezuela (Contreras-Ramos 1999, 2005).

*Species*	*Distribution*
Corydalus affinis Burmeister, 1839	Argentina, Bolivia, Brazil, Colombia, Ecuador, French Guiana, Guyana, Paraguay, Peru, Venezuela
Corydalus armatus Hagen, 1861	Argentina, Bolivia, Colombia, Ecuador, Peru, Venezuela
Corydalus arpi Navás, 1936†	Brazil, Venezuela
Corydalus batesii MacLachlan, 1868	Bolivia, Brazil, Colombia, Ecuador, French Guiana, Guyana, Peru, Suriname, Venezuela
Corydalus clavijoi Contreras-Ramos, 2002	Venezuela
Corydalus crossi Contreras-Ramos, 2002	Venezuela
Corydalus flavicornis Stitz, 1914	Colombia, Costa Rica, Ecuador, El Salvador, Guatemala, Honduras, Panama, Peru, Venezuela
Corydalus flinti Contreras-Ramos, 1998†	Venezuela
Corydalus hayashii Contreras-Ramos, 2002†	Venezuela
Corydalus hecate MacLachlan, 1866†	Brazil, Peru, Venezuela‡
Corydalus mayri Contreras-Ramos, 2002†	Venezuela
Corydalus neblinensis Contreras-Ramos, 1998	Venezuela
Corydalus nubilus Erichson, 1848	Brazil, French Guiana, Guyana, Venezuela
Corydalus peruvianus Davis, 1903	Argentina, Bolivia, Colombia, Costa Rica, Ecuador, Guatemala, Mexico, Panama, Peru, Venezuela
Corydalus tesselatus Stitz, 1914	Colombia‡, Venezuela
Corydalus wanningeri sp. n.	Venezuela

^†^ Male mandible short, female-like, with discrete dentition; ^‡^ Doubtful record.

## Methods

Isolated single specimens from a single site were collected by Prof. Wanninger, amounting to about 40 collections over a more than a 10 year span. All specimens were collected using mercury vapor light. The collecting site is located adjacent to the NE limit of Parque Nacional Canaima, in a portion of a winding road known as La Escalera (Spanish for ladder), highway 10, between Piedra de la Virgen and Danto Falls, around Km 110–112, at 1,000 m of elevation. A large communications antenna is a landmark for the collecting site. Highway 10 connects the Orinoco lowlands with the Gran Sabana plateau in the south. La Escalera is a humid slope covered with rain forest, with several brooks and waterfalls, potential habitat for the hellgrammites. Collections by Prof. Wanninger from nearby sites at higher and lower elevations did not produce any more specimens of the new species. Specimens were dissected and observed using standard techniques (Contreras-Ramos 1998).

Specimens will be deposited at Colección Nacional de Insectos, Instituto de Biología, UNAM, Mexico City (CNIN-UNAM), Museo del Instituto de Zoología Agrícola, Universidad Central de Venezuela, Maracay (MIZA), Zoologische Staatssammlung München, Bavaria, Germany (ZSM), and Prof. Wanninger’s private collection (RW). The identification key herein provided applies to species with males whose mandibles are elongate, with reduced dentition. Species with males having short, female-like mandibles may be identified with Contreras-Ramos (2002).

## Taxonomy

### Corydalus wanningeri

Contreras-Ramos & Von der Dunk sp. n.

urn:lsid:zoobank.org:act:D8CD9346-1591-4C3D-8898-A5145867FB18

**Figure 1.**
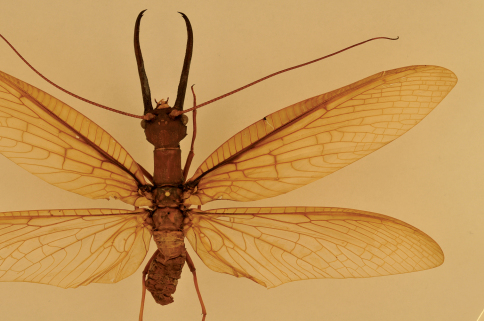
Corydalus wanningeri, sp. n. Male holotype, habitus.

Figures 1 [Fig F2] [Fig F3] [Fig F4] [Fig F5] [Fig F6] 8 

#### Etymology.

Named after Prof. Rupert Wanninger, amateur and outreach entomologist from Donaustauf, Bavaria, Germany, collector of the type series.

#### Type material.

Holotype, male, VENEZUELA: Bolívar, Escalera Km 110, el. 1,000 m, 26.viii.1994, leg. Rupert Wanninger [Head width 11.8 mm, mandible length 29.5 mm, antenna length 65.3 mm, forewing length 80.4 mm] (CNIN-UNAM). Paratypes: VENEZUELA, [Bolívar], Guyana, Km 120, 1997, 1 male [genitalia dissected] (MIZA); [Bolívar], Escalera, [Km 110, 1,000 m], 25.viii. 1999, [R. Wanninger], 1 female [genitalia dissected] (CNIN-UNAM); Bolívar, Escalera, Km 110, 1,000 m, 2.ii.2001, leg. R. Wanninger, 1 female (ZSM); [Bolívar, Escalera, Km 110, no date, R. Wanninger], 1 male (ZSM), 1 female (RW).

**Figure 2. F2:**
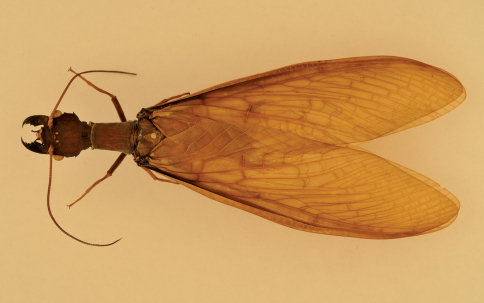
Corydalus wanningeri, sp. n. Female paratype, habitus.

#### Diagnosis.

**Figure 3. F3:**
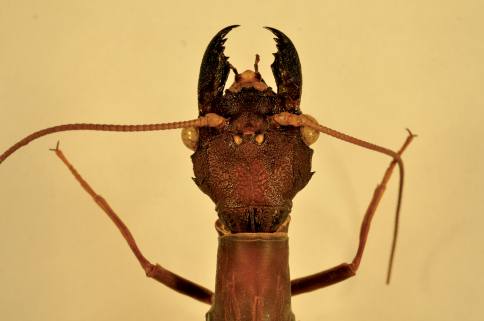
Corydalus wanningeri, sp. n. Female paratype, head and pronotum (dorsal).

**Figures 4–5. F4:**
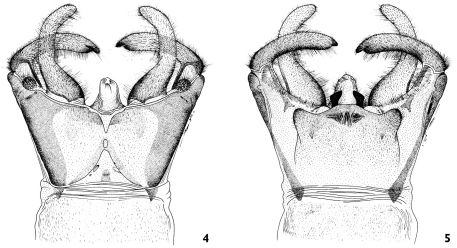
4 Corydalus wanningeri sp. n. **4** male genitalia (dorsal) **5** male genitalia (ventral).

**Figure 6. F5:**
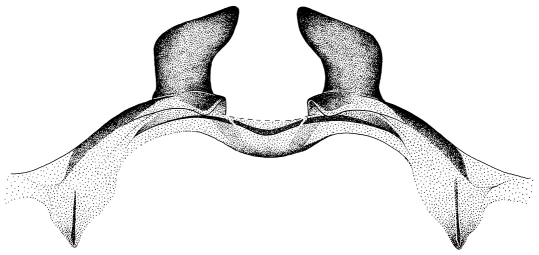
Corydalus wanningeri sp. n. Male tenth sternite.

Head and pronotum are reddish-brown, ferrugineous (Fig. 1–3), thus resembling in color species such as Corydalus cephalotes Rambur and Corydalus hecate MacLachlan, both with monomorphic mandibles (males with short mandibles with discrete dentition), or Corydalus batesii MacLachlan and Corydalus holzenthali Contreras-Ramos, with sexually dimorphic mandibles (males with elongate mandibles with reduced dentition). However, both color of body and wings most closely resembles Corydalus neblinensis Contreras-Ramos (Contreras-Ramos 1998, figs 124–127). In both species, antennae are paler than head and wings are pale reddish brown, unpatterned. However, in the new species ninth gonostyli are distinct (Figs 4, 5), with a narrowed apex (subclavate, unmodified in Corydalus neblinensis, Contreras-Ramos 1998, figs 26A, 26B). 10th sternite lobes (Figs 5, 6) are sclerotized, close to each other, convergent, and bluntly pointed (semimembranous, widely separated, and papilliform in Corydalus neblinensis, Contreras-Ramos 1998, fig. 26C). Females may be distinguished by the unpatterned reddish color and by a mandibular dentitional arrangement with an inner predental concavity and moderately separated first and second teeth (Figs 3, 8), similar to Corydalus nubilus and Corydalus crossi (Contreras-Ramos 1998, fig. 27I; Contreras-Ramos 2002, fig. 27).

#### Phylogenetic position.

Despite a close superficial resemblance to Corydalus neblinensis, Corydalus wanningeri sp. n., does not appear to be closely related to that or other kindred species, such as Corydalus batesii or Corydalus holzenthali. The new species appears to be closest to Corydalus crossi Contreras-Ramos, because of the structure of the 10th sternite and a basal protrusion of the 9th gonostylus. If so, the new species would be basal among species with elongate mandibles. At least, Corydalus wanningeri sp. n., should be basal with respect to species with a subclavate 9th gonostylus and non-incurvate 10th tergite, and so would lay out of Unnamed Group 1 (Contreras-Ramos 1998, table 36). However, a certain phylogenetic position of the new species would be known only after a formal phylogenetic analysis. Both Corydalus crossi and Corydalus wanningeri sp. n., share a Guayana Shield affinity.

#### Adult male.

**Figure 7. F6:**
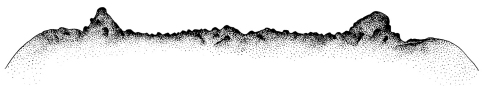
Corydalus wanningeri sp. n. Male clypeal margin (dorsal).

Head width 11.8–12.8 mm (average 12.3, *n* = 3), mandible length 29.5–30.3 mm (average 29.9 mm, *n* = 3), antenna length 64.5–65.3 mm (average 64.9 mm, *n* = 2), forewing length 77.7–80.4 mm (average 79.5 mm, *n* = 3), antenna length/forewing length 0.81–0.83. Color uniform dark reddish-brown. Head dark reddish-brown, unpatterned, mandible elongate with reduced dentition (Figs 1). Clypeal margin thinly black, lateral projections moderately developed, flat to slightly concave, median projection shallowly incised (Fig. 7). Antenna 87–89-segmented, filiform, scape and flagellum pale brown, tip infuscate. Maxilla blackish, 4-segmented palp brown. Labial palp 3-segmented, pale brown, last segment elongate.

Pronotum dark reddish-brown, unpatterned. Forewing pale reddish-brown, semitranslucent, unpatterned; veins reddish-yellow, except basal half of Sc and R infuscate; M1+2 3-branched (variably 4-branched), M3+4 a single vein. Hindwing pale-reddish, semitranslucent, basal 1/4 of R infuscate.

Genitalia (Figs 4–6). Ninth tergum subquadrate, V-shaped internal inflection reaching midlength of tergum. Anal tubercle without lateral sclerites. Tenth tergites slightly longer than ninth tergum, digitiform; basal 1/3 wide, roundly concave (Fig. 4). Ninth gonostylus subclavate, about 4/5 as long as 10th tergite, with narrowed digitiform apex (Fig. 5). Ninth sternum subquadrate, semimembranous, posterolateral lobes moderately developed (Fig. 5). Membrane between 9th and 10th sternites with thickened wrinkled portion. Tenth sternite moderately convex, anteromedian margin slightly convex; anterolateral projections moderately developed, blunt; lobes well sclerotized, elongate-trianguloid, apically convergent (Fig. 6). Pregenital sacs apparently absent.

#### Adult female.

**Figure 8.**
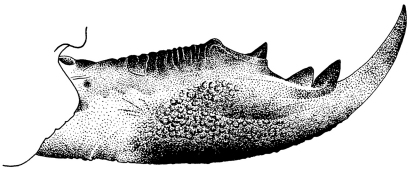
Corydalus wanningeri sp. n. Female mandible (right, dorsal).

Forewing length 65.1–80.6 mm (average 74.1 mm, *n* = 3). Color of body and wings similar to male (Figs 2, 3). Mandible with base dark reddish-brown, rest blackish; shape similar to Corydalus nubilus Erichson and Corydalus crossi, basal preapical tooth moderately separated from second (Fig. 8; Contreras-Ramos 1998, fig. 27I; Contreras-Ramos 2002, fig. 27). Antenna filiform, pale brown.

Terminalia non distinct. Sternal pouch between 6th and 7th abdominal segments well developed. Eighth sternum moderately sclerotized, discontinuous with pleural area, posterior margin mesally semimembranous, concave.

#### Key to long-mandibled males of known Corydalus species from Venezuela (modified from Contreras-Ramos 2002).

**Table:** 

1.	Ninth sternum modified, subattenuate and more sclerotized posteromesally (Contreras-Ramos 1998, fig. 27B) or with posteromedian projection (Contreras-Ramos 1998, fig. 31B)	2
–	Ninth sternum unmodified, subquadrate (Fig. 5; Contreras-Ramos 1998, figs 2B, 4B)	4
2.	Ninth sternum subattenuate, noticeably more sclerotized posteromesally (Contreras-Ramos 1998, fig. 27B)	Corydalus nubilus Erichson
–	Ninth sternum with posteromedian projection	3
3.	Posteromedian projection of 9th sternum large (nearly as long as sternum), thumblike; 9th gonostylus unguiform (Contreras-Ramos 1998, fig. 31B)	Corydalus tesselatus Stitz
–	Posteromedian projection of 9th sternum small (about 1/2 as long as sternum), narrow; 9th gonostylus tubular, with conspicuous preapical claw (Contreras-Ramos 2002, fig. 17)	Corydalus clavijoi Contreras-Ramos
4.	Ninth gonostylus elongate, somewhat flattened or tubular (Contreras-Ramos 1998, fig. 2B)	5
–	Ninth gonostylus subclavate (Fig. 5; Contreras-Ramos 1998, figs 4B, 7B, 17B)	6
5.	Ninth gonostylus and 10th tergite slender, subequal in length and shape (Contreras-Ramos 1998, fig. 2A)	Corydalus affinis Burmeister
–	Ninth gonostylus narrower and noticeably shorter than 10th tergite (Contreras-Ramos 2002, fig. 23)	Corydalus crossi Contreras-Ramos
6.	Head and pronotum reddish brown; 10th tergite apex without incurvation (Fig. 5), although it may be enlarged (Contreras-Ramos 1998, figs 7F, 26E)	7
–	Head and pronotum yellowish to greenish brown; 10th tergite with well developed apical incurvation (Contreras-Ramos 1998, figs 4A, 17B)	9
7.	Forewing contrastingly patterned (Contreras-Ramos 1998, fig. 58)	Corydalus batesii MacLachlan
–	Forewing not so contrastingly patterned	8
8.	Forewing pale, clear, nearly translucent, few subtle small white spots (Contreras-Ramos 1998, figs 124–126); 9th gonostylus unmodified, 10th sternite lobes papiliform, separated (Contreras-Ramos 1998, figs 26B, 26C)	Corydalus neblinensis Contreras-Ramos
–	Forewing rather opaque, uniformly pale reddish (Fig. 1); 9th gonostylus with narrowed digitiform apex, 10th sternite lobes elongate-trianguloid, close to each other (Figs 5, 6)	Corydalus wanningeri, sp. n.
9.	Antenna conspicuously subserrate, sinuate (Contreras-Ramos 1998, fig. 17F); 10th sternite with anteromedian projection (Contreras-Ramos 1998, fig. 17C)	Corydalus flavicornis Stitz
–	Antenna slightly subserrate; 10th sternite without anteromedian projection (Contreras-Ramos 1998, fig. 4C)	10
10.	Antenna, including scape and pedicel, pale to dark brown, apically infuscate (Contreras-Ramos 1998, figs 43, 44, 48); 10th sternite lobes typically subequal in width and length, less than half length of lobe surpassing posterior edge of 10th sternite (Contreras-Ramos 1998, fig. 4C); pregenital sacs well developed, conspicuous (Contreras-Ramos 1998, fig. 4F)	Corydalus armatus Hagen
–	Antenna, including scape and pedicel, yellow to yellowish green, up to distal 1/3 infuscate (Contreras-Ramos 1998, figs 139–141); 10th sternite lobes typically about twice as long as wide, about half of lobe surpassing posterior edge of 10th sternite (Contreras-Ramos 1998, fig. 29C); pregenital sacs apparently absent, inconspicuous	Corydalus peruvianus Davis

## Supplementary Material

XML Treatment for Corydalus wanningeri
